# Variations to the Nanotube Surface for Bone Regeneration

**DOI:** 10.1155/2013/513680

**Published:** 2013-04-28

**Authors:** Christine J. Frandsen, Karla S. Brammer, Sungho Jin

**Affiliations:** Materials Science and Engineering Program, University of California, San Diego, La Jolla, CA 92093-0411, USA

## Abstract

The complex mechanisms of the bone cell-surface interactions are yet to be completely understood, and researchers continue to strive to uncover the fully optimized implant material for perfect osseointegration. A particularly fascinating area of research involves the study of nanostructured surfaces, which are believed to enhance osteogenic behavior, possibly due to the mimicry of components of the extracellular matrix of bone. There is a growing body of data that emphasizes the promise of the titanium oxide (TiO_2_) nanotube architecture as an advanced orthopedic implant material. The review herein highlights findings regarding TiO_2_ nanotube surfaces for bone regeneration and the osteogenic effects of minute changes to the surface such as tube size and surface chemistry.

## 1. Characteristics and Function of Normal Bone

Bone is a complex tissue that has the ability to heal and regenerate itself [1], and is continuously in the cycle of remodeling from before birth until death [2]. The process of bone modeling and remodeling typically occurs to help the bone adapt to mechanical forces or to replace microdamaged bone with new, stronger bone [2]. Occasionally bone defects will form that are unable to heal on their own, either due to bone disease or trauma. In these cases, bone reconstruction is necessary, which requires osteoproduction (colonization of osteogenic stem cells at defect site), osteoinduction (induced bone formation), osteoconduction (growth of bone on a surface), osseointegration (stable anchorage of an implant achieved by direct bone-to-implant contact), mechanical stimulation, and vascularization [1, 3, 4]. In many cases an orthopedic implant is needed in order to stabilize the defect and provide support for new bone to grow. In order for orthopedic surgery to be successful, a strong and lasting connection between the implant and the interfacing bone tissue must be quickly established. A large part of current orthopedics research is centered on designing the material surface to more readily recruit bone forming cells to that interface.

## 2. The Evolution of Biomedical Materials Technology

The technology and design of materials for bone implants have evolved tremendously over the past 50 years, through what Hench and Polak defines as three generations of biomedical materials [5]. First-generation biomedical materials (of the 60s and 70s) were designed solely to “achieve a suitable combination of physical properties to match those of the replaced tissue with a minimal toxic response in the host” [5]. In the late 70s to early 80s, the focus of biomaterials design shifted from solely a bioinert tissue response to include a bioactive tissue response which would trigger a controlled reaction *in vivo* [5]. Bioactive materials reached clinical use by the mid of 1980s for orthopedic and dental applications, including bioactive glasses, ceramics, and composites [5]. Resorbable biomaterials (which dissolve into the body) were also introduced to the market, and resorbable polymer sutures became routinely used. At this point, biomaterials properties were beginning to be designed to match the body tissue at the microscale. The third generation of biomaterials has evolved as the search continues for an orthopedic implant technology that provides a stable osseointegration that routinely out-lives the patient. The aim of third-generation biomaterials is that the material is designed in such a way that it would stimulate certain cellular responses at the *molecular* level [5]. By modifying the surfaces at the molecular and nanoscale levels, researchers have been able to direct cell proliferation, differentiation, and extracellular matrix (ECM) production and organization [5]. Great progress has been made in the research behind third-generation biomaterials; however, the complex mechanisms of the bone cell-surface interactions are yet to be completely understood, and researchers continue to strive to uncover the fully optimized implant material for perfect osseointegration.

“Third-generation”, or nanostructured biomaterials research, has uncovered many interesting aspects of cell-surface interaction, and many believe that these materials will provide the optimal implant surfaces of the future. Although this review focused specifically on the nanotube surface architecture for bone regeneration, the reader is encouraged to read some interesting and informative literature on the more generic topic of nanostructured surfaces for osteogenesis, including [6–14].

## 3. Metal Oxide Nanotubes

Owing to attractive properties such as the high surface-to-volume ratios and size-dependent properties, nanostructured materials have been at the center of a large body of innovative research in science and technology. In particular, nanostructured surfaces are at the focal point of tissue engineering research due to findings which have demonstrated that cells will respond to and be directed by dimensions in the nanometer regime (<100 nm), even as small as 10 nm height [15, 16]. Although there are many methods for the fabrication of precisely defined nanostructured surfaces, one of the most simple and inexpensive processes for nanostructure formation is electrochemical anodization. Other common procedures for nanostructure fabrication involve a complicated series of steps which often can only be applied to a perfectly flat substrate (i.e., nanolithography). In contrast, electrochemical anodization can be applied to substrates of various 2D and 3D geometries and shapes, as well as sizes ranging from very small to potentially unlimited proportions. This method is also attractive because it is most commonly applied to titanium, which is one of the most widely used materials in bone implant technology. Consequently, the review herein is specifically concentrated on variations to the nanotube surface formed by electrochemical anodization in a fluorine containing electrolyte for orthopedic device applications.

The formation of titanium oxide (TiO_2_) nanotube arrays via electrochemical anodization was first reported by Gong et al. in 2001 [17]. Since their discovery, researchers have achieved better control of nanotube formation through such methods as varying electrolyte concentration and pH, inorganic and aqueous solvents, and so forth. Extensive control is now possible for nanotube morphology and dimensions including diameter, length, length-to-diameter ratio, wall-thickness, tube shape (conical versus cylindrical), transparency, and even doping [18]. Such ability to precisely control nanotube formation via electrochemical anodization has great promise for their utilization in the orthopedics industry.

In addition, researchers have been able to apply the same technique of electrochemical anodization to other metals, including zirconium (Zr) [19] and tantalum (Ta) [20] thus forming nanotube arrays of these metal oxides. The work discussed in this review will focus solely on titanium oxide (TiO_2_) and zirconium oxide (ZrO_2_) nanotubes, as well as variations to the TiO_2_ surface and corresponding effects on osteoblast and mesenchymal stem cell growth and function.

**Figure 1:**
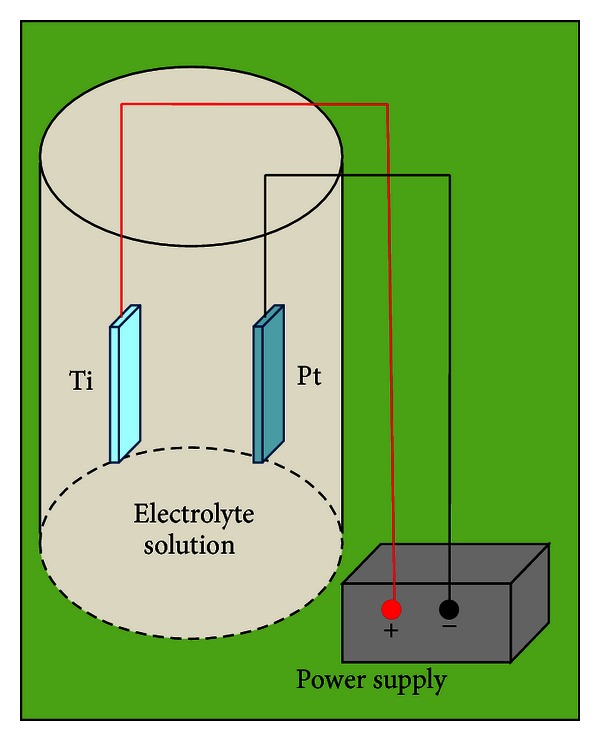
Schematic drawing of the electrochemical anodization setup. Fabrication variables include voltage, electrolyte concentration, temperature, and pH.

Electrochemical anodization of titanium (Ti) or other metal foils involves a two-electrode electrochemical cell with a platinum (Pt) foil cathode and metal foil (titanium or other metal of choice) anode which are held at a constant potential (see Figure 1). The traditional method of anodization utilizes a hydrofluoric-acid-(HF-) based electrolyte; however, researchers have also used ammonium fluoride and other chemicals as the fluorine ion source. Additionally, the original electrolyte solutions were aqueous based; since then, it has been found that increased control of nanotube morphology can be achieved using inorganic solvents [21], and increased mechanical robustness can be achieved using the addition of acetic acid to the HF electrolyte in a 1 : 7 ratio [18]. A trend in nanotube growth, that is, consistent, no matter the electrolyte concentration, is increasing nanotube diameter with increasing applied voltage. The reader is directed to a thorough review by Mor et al. for a detailed mechanistic model of nanotube formation by electrochemical anodization [18].

## 4. Osteoblast and Mesenchymal Stem Cell Growth and Functionality on TiO_**2**_ Nanotubes

**Figure 2:**
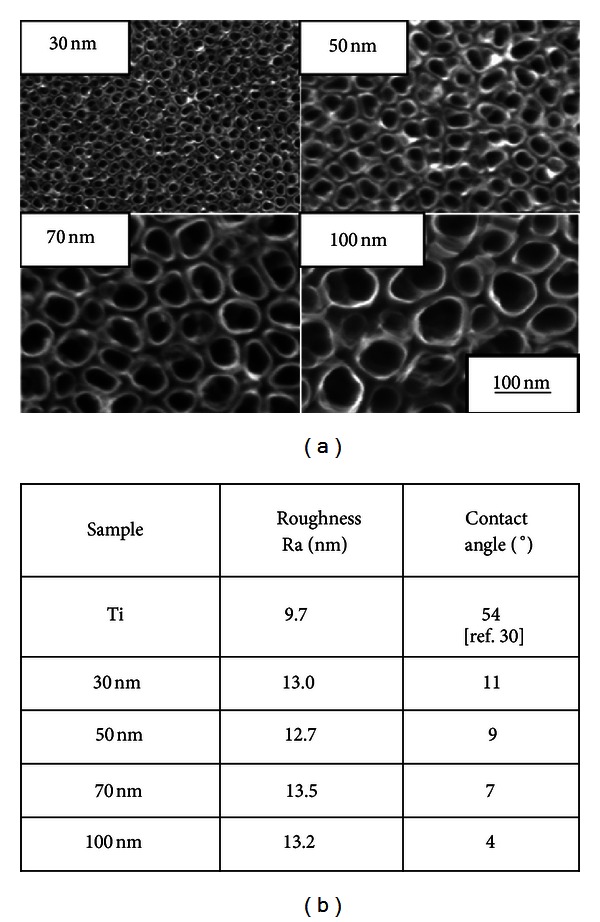
Physical characterization of different-sized nanotube surfaces. (a) SEM micrographs of self-aligned TiO_2_ nanotubes with different diameters. The images show highly ordered nanotubes with four different pore sizes between 30–100 nm. (b) Table with average roughness (*R*_*a*_) and surface contact angle measurements for Ti and 30–100 nm TiO_2_ nanotube surfaces.

In 2006, TiO_2_ nanotubes were demonstrated by Oh et al. to significantly accelerate osteoblast adhesion and proliferation and enhance bone mineral formation when compared to nonmodified titanium surfaces [22]. These initial experiments were performed on TiO_2_ nanotubes of  ~100 nm diameter and ~300 nm height, with a wall thickness of  ~10 nm. Popat et al. reported similar observations in 2007 on the higher adhesion, proliferation rates, and bone forming ability of marrow stromal cells on TiO_2_ nanotoubular surfaces (80 nm diameter, 400 nm height) when compared to those grown on flat titanium surfaces [23]. The intriguing phenomena in these studies of differing cell types triggered further investigation into the effects of nanotube geometry on osteogenic behavior. As was mentioned previously, precise control of the nanotube diameter is possible by varying the applied voltage during anodization. In order to further understand the influence of the nanotube architecture on bone cell behavior, a series of studies were performed in which the lateral spacing of the nanotube system was varied by altering the nanotube diameter from 30, 50, 70, and 100 nm, as depicted in Figure 2. The average surface roughness (Ra) and contact angle measurements for the corresponding flat Ti and various nanotube surfaces are reported in Figure 2(b).

## 5. Effect of Nanotube Size on Osteogenic Behavior

**Figure 3:**
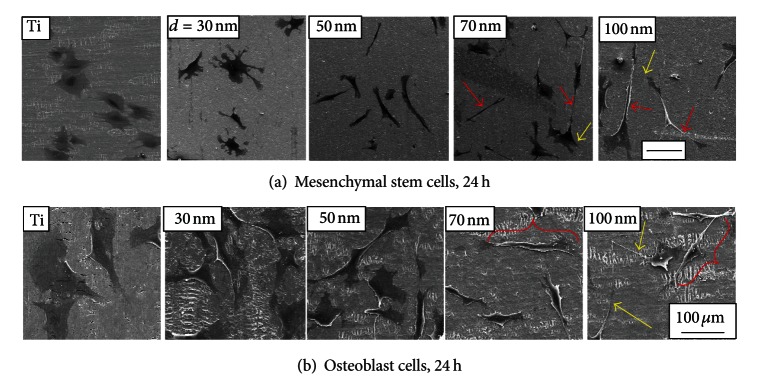
SEM micrographs of (a) human mesenchymal stem cells (hMSCs) and (b) osteoblast cells cultured on flat Ti and 30, 50, 70, and 100 nm diameter TiO_2_ nanotube surfaces after 24 h of culture (scale bar, 100 *μ*m). Red arrows (a) and brackets (b) emphasize extraordinary cell elongation; yellow arrows indicate elongated leading edges of lamellipodia.

Most bone implant materials are placed in direct contact with both adult bone and bone marrow tissue and thus are exposed to two main cell types: osteoblasts (bone cells) and mesenchymal stem cells (bone marrow cells). In order to develop an understanding of the role of the TiO_2_ nanotube surface *in vitro*, the behavior of osteoblast cells and mesenchymal stem cells (MSCs) was studied on a series of nanotube sizes shown in Figure 2. Experimental conditions and sample preparation techniques were held constant in both studies, while only the cell type was varied. The cell morphology of both osteoblast and MSCs were analyzed using scanning electron microscopy (SEM). In both studies, an increase in cell elongation was observed as a function of nanotube diameter, as shown in Figure 3. On the flat Ti substrate, both cell types are flat, spread out, and round-shaped; they are somewhat flat and rounded on 30 nm nanotubes, and they become progressively elongated as the nanotube diameter is increased to 50 nm diameter and beyond. It is evident that the nanotubes with diameters of 70 and 100 nm induce extraordinary cell elongation (see red arrows and brackets) after 24 h of culture. In addition, the elongated leading edges of lamellipodia (yellow arrows) of both cell types indicate that the cell morphologies are more mobile on the 70 and 100 nm nanotubular surfaces.

**Figure 4:**
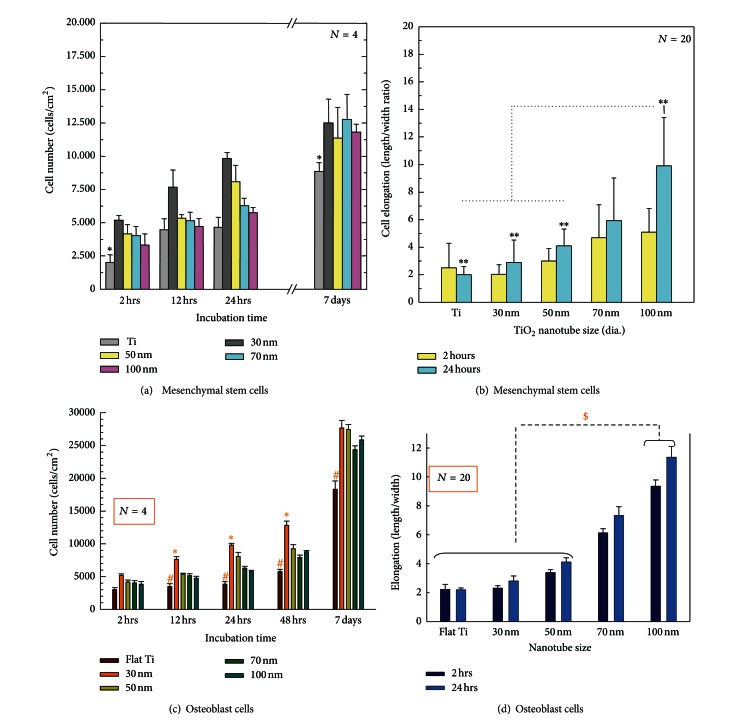
Comparative graphs showing the influence of nanotube diameter on cell number and elongation at early incubation time points. MSC cell number versus incubation time (a) and MSC elongation (length to width ratio) as a function of nanotube diameter at 2 and 24 h (b). Osteoblast cell number versus incubation time on each substrate (c) and osteoblast cell elongation as a function of nanotube diameter at 2 and 24 h (d).

The number of cells that adhered to each surface was measured as a function of incubation time. The results of the MSC study are shown in Figure 4(a), and the results of the osteoblast study are shown in Figure 4(c). The highest number of adhered cells in both studies was found on the 30 nm diameter nanotube surface. In addition, the cell elongation of both experiments was quantified by calculating the ratio of cell length to width; the data of the MSC experiment is shown in Figure 4(b), and the data from the osteoblast experiment is shown in Figure 4(d). As was observed in the SEM images in Figure 3, both cell types become increasingly elongated as the nanotube pore size increases. However, comparing the cell adhesion versus the cell elongation in Figure 4, it is apparent that these phenomena follow opposite trends as a function of nanotube diameter.

**Figure 5:**
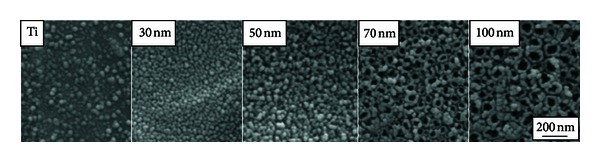
SEM micrographs showing protein adsorption on the surfaces of flat Ti and 30, 50, 70, and 100 nm diameter TiO_2_ nanotubes after 2 h of incubation in growth medium.

The results of the osteoblast and MSC cell studies indicate that the nanotube dimensions play an important role in the initial cell response to the surface, as indicated by the cell adhesion and elongation behaviors. The mechanism through which a cell senses and attaches to a surface is through surface receptors called integrins, which in fact do not sense the surface, but proteins adhered to the surface. Thus, in order to understand the behavior of cells on the nanotube topography, it is important to investigate the manner with which proteins are adsorbed onto the substrates.  Figure 5 shows scanning electron microscope (SEM) images of proteins adsorbed onto the flat Ti and 30, 50, 70, and 100 nm diameter nanotube surfaces after 2 hours of incubation in cell culture medium. While the presence of protein aggregates is infrequent on Ti, there is an abundance of aggregates on the 30 nm nanotubes. However, the proteins on the larger diameter 70 and 100 nm nanotubes are few and are spaced farther apart. It is evident from these micrographs that the nanotube diameter causes distinct differences in the number and placement of proteins on the surface.

The phenomena of cell adhesion versus elongation on the nanotube surfaces can be explained by the pattern of protein adsorption on each of the substrates. The small pore size of the 30 nm surface allows for proteins to adsorb in a more tightly knit fashion, which enables the cells to adhere easily. Additionally, the 30 nm substrate does not direct the cells to move/stretch in any specific direction since proteins are everywhere. In contrast, the placement of proteins on the larger (70 and 100 nm) nanotube topographies encourages cell spreading due to the adsorption of proteins only on the nanotube wall rims, thus inducing a fixed distance between proteins and encouraging the cell to spread in order to find adhesion proteins. It is probable that the cells are required to expand their filopodia across larger distances, thus inducing the elongated cell shape. This would also affect the ability of the cell to adhere to the surface, which explains the lower number of adhered cells on the larger diameter substrates.

**Figure 6:**
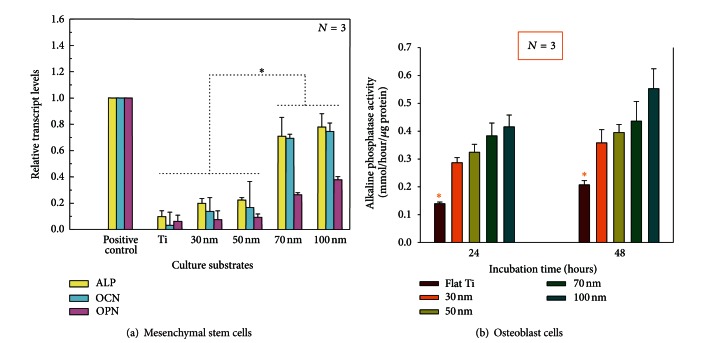
Comparative graphs showing the trend of MSC and osteoblast cell functionality with increasing nanotube diameter. (a) Quantitative PCR analysis for ALP, OCN, and OPN after 3 week mesenchymal stem cell culture. Plastic cell culture plate with osteogenic inducing media was used as a positive control for osteogenic differentiation. (b) ALP activity after 24 and 48 h of osteoblast incubation.

In addition to the observations of cell adhesion and elongation, the MSC and osteoblast cell behavior were also analyzed in terms of bone-forming functionality. In the MSC study, quantitative polymerase chain reaction (PCR) analysis was performed in order to estimate the relative transcript levels of alkaline phosphatase (ALP), osteocalcin (OCN), and osteopontin (OPN) gene expressions. The presence of these three genes is important because ALP is an enzyme produced by cells which indicates their bone-forming ability, while OCN and OPN are proteins found in bone. The data from the PCR analysis, shown in Figure 6(a), demonstrates significantly higher gene expression of all three genes on the larger diameter (70 and 100 nm) substrates when compared to the flat Ti and smaller diameter (30 and 50 nm) substrates, indicating osteogenic differentiation. Similarly, alkaline phosphatase activity of the osteoblast cells was measured on each of the experimental culture substrates. The results from the osteoblast study portray the same trend of increasing ALP activity with increasing nanotube diameter (Figure 6(b)). These results are evidence that the nanotube diameter causes an upregulation in the markers of bone formation.

Since the cell elongation and cell functionality followed the same increasing trends as a function of nanotube diameter, it can be speculated that there is a correlation between the two phenomena. Interestingly, in addition to the highly elongated cell shape on the large diameter nanotube surfaces, the cell nuclei on these surfaces were also elongated (by 20–25%, data not shown). It is likely that the elongation of the cell nuclei is a result of the stretching of the cytoskeletal morphology of the cell. Researchers have indicated that cytoskeletal reorganization can cause nuclei distortion, which may promote differences in DNA behavior due to mechanical restraints within the nuclei [24, 25]. Therefore, it is evident that the large diameter nanotube substrate induces cell elongation and thus nuclei distortion, which may cause osteoblast and MSCs to produce markers of bone formation and osteogenic differentiation more readily than on a flat substrate.

**Figure 7:**
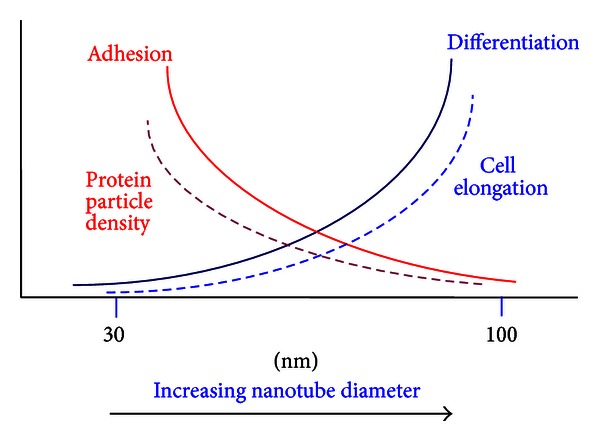
Schematic illustration of the overall trends of nanocue effects on cell fate and morphology. The change in cell adhesion and growth without differentiation (solid red line) has the same trend as protein particle density (broken red line), whereas that of differentiation (solid blue line) has the same trend as cell elongation (broken blue line).

The overall trends of the nanocue effects on osteoblast and stem cell morphology and fate can be summarized by the schematic illustration in Figure 7. It was observed that with increasing nanotube diameter cell adhesion growth decreased (solid red line), in a similar manner as protein particle density (broken red line). In contrast, both osteoblast and MSCs demonstrated a higher degree of osteogenic differentiation (solid blue line) with increasing nanotube size, analogous to the trend of cell elongation (broken blue line).

The findings of these two studies give light to increased understanding of the role of nanostructure dimensions for enhanced biomaterial surface design. However, the nanotube size in these studies was restrained to a maximum diameter of 100 nm due to the limitations of _TiO2_ anodization in an aqueous hydrofluoric acid electrolyte as was used for preparation of these surfaces. Anodization methods that enable the fabrication of larger diameter nanotube arrays have been reported, even to as large as 350 nm diameter using an electrolyte consisting of diethylene glycol with low concentrations of hydrofluoric acid (HF) [26]. With careful control of anodization protocols, eventual progress may be made which enables the growth of mechanically strong nanotubes with large diameter pore openings. It would be of great interest to further investigate the osteoblast and MSC behaviors at diameters beyond 100 nm.

The size effect of the nanodimensions of TiO_2_ nanotube surfaces raises the question of whether the trend is unique to the TiO_2_ nanoarchitecture fabricated via electrochemical anodization or if osteogenic behavior would be similar on various sizes of nanotubes of different surface chemistries. In 2009 an interesting study was published by Bauer et al. in which the size selective behavior of MSCs was analyzed on ZrO_2_ nanotubes as well as TiO_2_ nanotubes coated with a conformal layer of AuPd [27]. Brammer et al. observed that the different surface chemistries did not affect the diameter dependence of cell adhesion or proliferation. However, a more recent study by the Jin lab has demonstrated that various surface chemistries do affect multiple cell types differently [28], as will be described in detail in the following section. 

## 6. Variations of Nanotube Surface Chemistry

Although nanostructured surface geometries have provided exciting findings in the latest biomaterials research, only a few publications have directly compared nanostructures of various surface chemistries [28]. Usually, the surface chemistry is held constant, while the nanotopography or nanogeometry is varied. The history of orthopedic implant materials has made it obvious that body tissues respond differently to surfaces depending on the type of foreign material [29]. Surface chemical factors are in fact one of the most significant factors on the nanoscale that can affect cell-material interactions [30]. Though the majority of related studies compare only nanotextured with nontextured surfaces of the same material, an important addition to this research would be the direct comparison of the same nanostructure with different surface chemistries. It is possible that a unique combination of surface chemistry and nanostructured geometry may provide a balance of defined characteristics towards an optimized cell response.

The advantages of the TiO_2_ nanotube surface topography for orthopedic applications have been well outlined in prior sections. Since titanium is one of the most commonly used orthopedic materials in use today, it is of great interest to compare any future materials with a well-recognized industry standard. Therefore, experiments in the Jin lab were designed to provide a direct comparison of flat Ti and TiO_2_ nanotubes with the other potential surface chemistries to advance orthopedic implant technology. In order to accomplish this, the concept was implemented of maintaining constant nanotube geometry (i.e., TiO_2_ nanotubes with 100 nm diameter as shown in Figure 2(a)), while varying the surface chemistry. 

## 7. Carbon Chemistry Effects on Osteogenic Behavior

Carbon films deposited on metal in both its amorphous and crystalline forms have been investigated as potential biomedical materials, mainly because of the chemical inertness of carbon and its naturally occurring presence in the human body [31–33]. The application of carbon films to materials that are sensitive to wear, such as Ti and Si, has been a convenient method that has shown significant potential for implant coating applications [34–40], specifically for orthopedic implants. The effect of a carbon thin film coating on the surface of TiO_2_ nanotubes is thus of interest and will be discussed in this section.

The nanotube substrates used in this study were prepared according to standard anodization protocol for the formation of TiO_2_ nanotubes with 100 nm diameter and 1 : 3 aspect ratio as shown in the bottom right corner of Figure 2(a). For the carbon-coated comparative surface, the TiO_2_ nanotubes were deposited with a thin, conformal layer of carbon by DC sputter deposition methods in order to obtain a nanotube architecture with a carbon surface chemistry. The carbon coating was only deposited in a very thin layer onto the nanotube wall rims, not altering the nanotube architecture in any way.

 Since cell behavior varies depending on cell type, both osteoblast and osteoprogenitor (mesenchymal stem) cells were plated in this study onto the experimental surfaces in order to assess their behavior in response to the surface chemistry/nanostructure combination. At early incubation time points, the cellular behavior on the surface *in vitro *includes cell adhesion, growth, and morphological orientation/organization. These three behaviors were assessed via MTT analysis, immunofluorescent cytoskeletal actin staining, and SEM examination (data not shown, the reader is directed to ref [28] for full details). The results of each of these assays for both osteoblast and osteoprogenitor cells at early time points (24 and 48 hours) were insignificantly different, which indicates that the carbon versus TiO_2_ surface chemistry did not affect the initial cell response to the surface. Both cell types adhered and proliferated equally well on both the TiO_2_ nanotube and carbon-coated nanotube surfaces. In addition, the cell morphology showed no difference.

The ability of osteoblasts and MSCs to mature properly and readily is a vital part of measuring cellular response for bone implant purposes. In this study, the two cell types were cultured for 3 weeks in order to analyze the cells function over time and to determine whether the surfaces were inhibiting or promoting bone function. It should be clarified that the experiments included in this study included the corresponding flat TiO_2_ and carbon substrates as control surfaces. The nanotube substrates were found to enhance both osteoblast and MSC cellular response when compared to the flat controls in all aspects of this study, and the data was thus not included.

**Figure 8:**
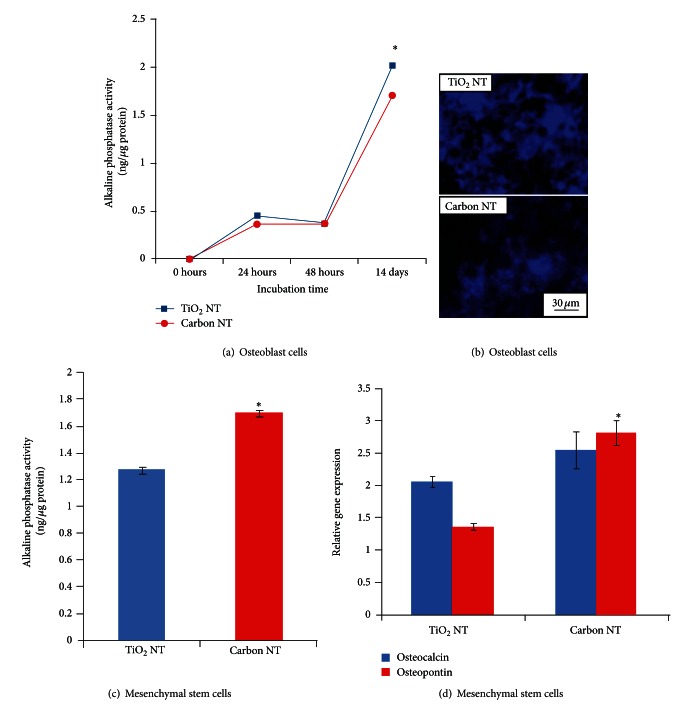
Comparison of the degree of osteoblast functionality and MSC differentiation and maturation in late stage culture (3 weeks) on the TiO_2_ versus carbon chemistry nanotube surfaces. ALP activity for osteoblasts (a) and MSCs (c) cultured on the nanotube surfaces shows different favorable chemistries. (b) Fluorescent images showing ALP staining of osteoblast cells verifying data shown in (a). (d) Quantitative PCR analysis of the MSCs on each surface for osteocalcin and osteopontin, verifying the trend in (c).

In order to assess the behavior of the osteoblast cells, the alkaline phosphatase (ALP) activity was measured, which is an enzyme indicative of bone forming ability. Comparative levels of ALP activity on each substrate are presented in Figure 8(a) as a function of incubation time. In order to visually verify the ALP activity quantitative results, the osteoblast cells were also stained using an ALP staining kit, as shown in Figure 8(b). The ALP activity indicates that the bone-forming ability of the osteoblast cells was enhanced on the TiO_2_ surface chemistry when compared to the ALP levels on the carbon surface.

In contrast, the MSCs appear to favor the carbon surface chemistry, as indicated by the enhanced ALP activity of the MSCs on the carbon NTs shown in Figure 8(c). Additionally, the degree of osteogenic differentiation and maturation of the MSCs was analyzed by quantitative PCR analysis for osteocalcin (OCN) and osteopontin (OPN). OCN and OPN are two major noncollagenous protein components of bone extracellular matrix which are considered markers of osteogenic differentiation as they are solely synthesized and secreted by osteoblastic cells. The graphs of relative amounts for each assay are shown in Figure 8(d); the relative gene expression of both proteins was enhanced on the carbon surface chemistry when compared to the TiO_2_ chemistry, and the OPN was especially upregulated. It is evident from these findings that the carbon surface chemistry causes an increase in osteogenic differentiation and function of the MSCs. These results indicate that MSCs can distinguish between surface chemistries and crystallinity, as other research groups have also observed [41].

The results of this study indicate that mesenchymal stem cells and osteoblast cells respond differently to remarkably different surface chemistries and seem to have different chemical preferences for optimal cell function. While osteoblast cells are mature bone cells specific to bone tissue, mesenchymal stem cells are highly sensitive, unprogrammed cells and are readily influenced by extracellular factors such as chemical and topographical cues. Therefore, it is not surprising that the two cell types have different preferences of surface chemistry. It is possible that the inclination of the MSCs for the C-coated surface may be explained by the fact that bone marrow contains many organic carbon-rich components. In contrast, bone tissue is composed of more ceramic/mineral rich components, similar to TiO_2_. Perhaps the different cell types are partial to distinct chemistries because of the chemical components of their natural extracellular environments *in vivo*.

## 8. Conclusions and Future Directions

It is apparent that TiO_2_ nanotubes as an advanced biomaterial are capable of strongly affecting cell behavior and that even minute changes in the nanotube surface can have substantial results. The fact that such a small range of dimensions as 30–100 nm diameter pore openings can alter cell functionality has great promise for researchers in the field of bone regeneration. Additionally, since the process of electrochemical anodization provides such facile methods for altering nanotube dimensions, and is applicable to substrates of 2- and 3D geometries, it is imaginable that these findings would be appealing. Furthermore, the differing cellular preferences for various surface chemistries indicate the potential for further experiments comparing multiple surface chemistries with the same underlying nanotopography. Unique combinations of topography and chemistry could provide the ability to tailor a medical implant surface to a particular tissue type, which would revolutionize the field of regenerative medicine.
